# Overview of Global Trends in Classification, Methods of Preparation and Application of Bacteriocins

**DOI:** 10.3390/antibiotics9090553

**Published:** 2020-08-28

**Authors:** Maria Zimina, Olga Babich, Alexander Prosekov, Stanislav Sukhikh, Svetlana Ivanova, Margarita Shevchenko, Svetlana Noskova

**Affiliations:** 1 Institute of Living Systems, Immanuel Kant Baltic Federal University, A. Nevskogo Street 14, 236016 Kaliningrad, Russia; mariia.zimina@list.ru (M.Z.); olich.43@mail.ru (O.B.); stas-asp@mail.ru (S.S.); lionsorciere@gmail.com (M.S.); svykrum@mail.ru (S.N.); 2 Laboratory of Biocatalysis, Kemerovo State University, Krasnaya Street 6, 650043 Kemerovo, Russia; a.prosekov@inbox.ru; 3 Department of Bionanotechnology, Kemerovo State University, Krasnaya Street 6, 650043 Kemerovo, Russia; 4 Natural Nutraceutical Biotesting Laboratory, Kemerovo State University, Krasnaya Street 6, 650043 Kemerovo, Russia; 5 Department of General Mathematics and Informatics, Kemerovo State University, Krasnaya Street, 6, 650043 Kemerovo, Russia

**Keywords:** bacteriocins, antimicrobial action, cytotoxic action, activity of bacteriocins, probiotics, PCR reactions

## Abstract

This paper summarizes information about the division of bacteriocins into classes (Gram-negative bacteria, Gram-positive bacteria, and archaea). Methods for producing bacteriocins have been studied. It is known that bacteriocins, most successfully used today are products of secondary metabolism of lactic acid bacteria. It is established that the main method of bacteriocin research is PCR analysis, which makes it possible to quickly and easily identify the presence of bacteriocin encoding genes. The mechanism of cytotoxic action of bacteriocins has been studied. It is proved that the study of cytotoxic (antitumor) activity in laboratory conditions will lead to the clinical use of bacteriocins for cancer treatment in the near future. It is established that the incorporation of bacteriocins into nanoparticles and targeted delivery to areas of infection may soon become an effective treatment method. The delivery of bacteriocins in a concentrated form, such as encapsulated in nanoparticles, will increase their effectiveness and minimize potential toxic side effects. The analysis of publications on this topic confirmed that diverse research on bacteriocins is relevant.

## 1. Introduction

Bacteriocins are ribosomally synthesized proteins or short polypeptides that have antibacterial activity and are closely related to the producing strain. Bacteriocins act by interacting and destroying cells with specific surface receptors [1]. In a microbial community, cells can be bacteriocinogenic (produce bacteriocin), sensitive, or resistant to each bacteriocin. When all three cell types are present and compete for limited resources, only a small percentage of bacteriocinogenic cells will be induced to produce and release bacteriocin [2]. Some sensitive cells are immediately destroyed, while others contain mutations that determine their resistance. Resistant cells quickly displace producing cells due to the bacteriocin production “cost” [3].

Bacteriocin encoding genes, a set of immune proteins, and other auxiliary proteins are organized into operons that are located in the ring chromosome, plasmids, or other mobile genetic elements [4]. These gene clusters are usually inducible and require the secretion and extracellular accumulation of peptides for induction [5,6,7,8].

Bacteriocins are antibacterial peptides synthesized by ribosomes produced by bacteria that inhibit the growth of similar or closely related bacterial strains [9]. A number of bacteriocins from a wide variety of bacteria are discovered and their various structures are described. More and more data indicate that bacteriocins have different structures, modes of action, mechanisms of biosynthesis and self-immunity, as well as gene regulation. Bacteriocins are considered an attractive compound to use in the food and pharmaceutical industries for preventing food spoilage and the growth of pathogenic bacteria. Moreover, the elucidation of their biosynthesis led to the use of gene expression systems controlled by bacteriocin and enzymes of biosynthesis of lantibiotics, a class of bacteriocins, as tools for the creation of new peptides [9].

A huge variety of chemical structures allows bacteriocins to affect various vital functions of a living cell (transcription, translation, replication, and cell wall biosynthesis), but most act by forming membrane channels or pores that violate the energy potential [2]. Studies of the modes of action of bacteriocins focus on two different action aspects—kinetics of the physical interaction between bacteriocin and susceptible cells and detection of specific biochemical lesions in target cells [10]. It is widely hypothesized that the interaction of bacteriocin with the target cell occurs in two stages. The first stage, which is probably reversible, corresponds to the physical adsorption of bacteriocin in the cell using receptors. Removing the bacteriocin at this stage appears to leave the cell intact, since there is no permanent physiological damage. The second stage leads to irreversible pathological changes as a result of specific biochemical damage to the cell [2].

It is known [11] that, in nature, microorganisms can have several mechanisms for establishing interaction and protection. One of these mechanisms is associated with the production of bacteriocins, peptides with antimicrobial activity. Bacteriocins are found in Gram-positive and Gram-negative bacteria. However, bacteriocins produced by Gram-positive bacteria are of particular interest, due to the industrial use of several strains belonging to this group, especially lactic acid bacteria (LAB), which have the generally recognized as safe (GRAS) status. This paper discusses the latest trends in the field of inventions and the state of the art related to the production of bacteriocin by Gram-positive microorganisms. Since 1965, one hundred and eight patents relating to manufacturers of Gram-positive bacteriocins have been filed, 57% of which are related bacteriocins derived from *Lactococcus, Lactobacillus, Streptococcus*, and *Pediococcus strains*. Surprisingly, patents for the production of heterologous bacteriocins have been presented mainly only in the last decade. Although the main application of bacteriocins is in the food industry to combat spoilage and foodborne bacteria, in recent years the use of bacteriocin has been shifted to the diagnosis and treatment of cancer, as well as resistance to plant diseases and growth stimulation [11].

In [12], it is described that bacteriocins are ribosome-synthesized (poly) peptides produced by almost all prokaryotic lines. Bacteriocins from lactic acid bacteria and probiotic bacteriocin-producing organisms have been thoroughly studied due to their broad spectrum of action, long-term use in food fermentation, and consideration of these microorganisms as beneficial to humans. Most research on the biotechnological use of various bacteriocins has focused on their use as food preservatives, the prototype of which is nisin, which has been successfully used in food. However, bacteriocins from LAB have demonstrated remarkable potential as therapeutic agents for medical or veterinary use, alone or in combination with conventional antimicrobial agents. The interest in them is even greater now, when resistance to antibacterial agents used in therapy is growing [12].

It is expected [13] that the bacteriocins produced by lactic acid bacteria will be safe antimicrobial agents. While the most studied LAB bacteriocin, nisin A, is widely used as a food preservative, various new bacteriocins are required to better control unwanted bacteria. In order to detect new bacteriocins at an early stage of the screening process, a rapid screening system that evaluates the bacteriocins produced by recently isolated LABs, based on their antibacterial spectra and molecular weights is developed. Various new bacteriocins have been identified using this system, including nisin variant, nisin Q, two-peptide bacteriocin, lactococcin Q, leaderless bacteriocin, lacticin Q and ring bacteriocin, lactocyclic Q. Moreover, it is found that some LAB isolates produce several bacteriocins [13]. They have been characterized in relation to their structure, mechanisms of action and biosynthetic mechanisms. The new LAB bacteriocins and their biosynthetic mechanisms are used for such applications as food preservation and peptide engineering.

Bacteriocins—bacterial proteins or peptides—are considered as candidates for a new generation of effective antimicrobial agents [14]. The analysis of the properties of natural and genetically modified bacteriocinosis from the standpoint of the molecular basis of their production and activity is presented. Most bacteriocins have a narrow spectrum of inhibitory activity. Some of the broad-spectrum bacteriocins have a ring structure (the C- and N-ends of the amino acid chain are linked by a peptide bond). The fixed position of the ends of the protein molecule gives the protein the ability to bind to different receptors on the surface of target cells. Genes encoding the production of bacteriocins and associated proteins can be expressed in foreign cells, including eukaryotic cells. There is also information on changes in the properties of bacteriocin by using site-specific mutagenesis [14].

Bacteriocins are small peptides with antibacterial properties. They are produced by both Gram-positive and Gram-negative bacteria. To date, several hundred bacteriocins have been described [15]. The classification of bacteriocins is undergoing constant changes as new developments appear regarding their structure, amino acid sequence, and the recognized mechanism of their action. Some bacteriocins (lantibiotics) contain atypical amino acids such as lanthionine (Lan), methylanthionine (MeLan), dehydroalanine (Dha), dehydrobutyrin (Dhb), or D-alanine (D-Ala). The best known bacteriocins are produced by lactic acid bacteria, including nisin, produced by strains of *Lactococcus lactis*. These bacteriocins are recognized as completely safe for humans. Nisin is currently used in the food industry as a preservative [16].

The purpose of this work is to analyze global trends in bacteriocin research (classification, description and comparison of methods for their production, application, etc.). The cytotoxic potential of bacteriocins has been established and the need for an in-depth study of the cytotoxic mechanism of action of modern bacteriocins has been shown.

The study is relevant, since it systematizes information related to the detection of specific biochemical lesions in cells using bacteriocins and possible methods of combating these lesions. 

## 2. Classification of Bacteriocins

### 2.1. Cell Type Classification

The classification of bacteriocins is based on the cell wall type of producing organisms (Gram-negative and Gram-positive). To date, several bacteriocins produced by the *Archaea domain reprsentatives* (Table 1) have also been characterized.

Although the adsorption of bacteriocins is highly specific in many cases, some bacteriocins, such as staphylococcins 414 and 1580, lactocin LP27 and streptococcin B-74628, do not have specific adsorption. Each of these bacteriocins is absorbed by bacteria resistant to its effects. This interaction may be due to the high surface activity of the compounds [29,30,31].

#### 2.1.1. Bacteriocins from Gram-Negative Bacteria

Bacteriocins of Gram-negative bacteria are divided into four main classes: colicins, colicin-like, phage-tail-like bacteriocins, and microcins [17]. Colicins are protease-sensitive, heat-sensitive high-molecular weight (30–80 kDa) bactericidal proteins synthesized by most *E. coli* strains that have a single colicinogenic plasmid. Compounds of this class are the most studied and are used as model systems for studying the structures, functions, and evolution of bacteriocins [22]. Colicin synthesis is carried out under stress and is fatal for producing cells, due to co-expression with lysis protein [32]. Depending on the mechanism of interaction with the target cell, colicins are divided into three main groups—pore-forming, nuclease, and peptidoglycan degrading. Colicin uptake by the target cell is accomplished by receptors involved in the transport of nutrients such as vitamin B12 (cobalamin receptor BtuB), siderophore FhuA-, FepA-, Cir-, and Fiu-bound iron and nucleosides (Tsx receptor). In addition, some colicins use porin proteins that control the passive diffusion of sugars, phosphates, and amino acids through the outer membrane [33]. Protein bacteriocins produced by other Gram-negative bacteria are classified as colicin-like due to the presence of similar structural and functional characteristics [34]. The central R-domain is involved in binding to the receptor, the N-terminal T-domain is responsible for translocation and provides penetration of bacteriocin into the cell, while the C-terminal C-domain is the active center of bacteriocin and causes its cytotoxicity [34].

Phage tail-like bacteriocins (tailocins) are larger protein structures (20–100 kDa) consisting of eight to fourteen different polypeptide subunits that show similarities to the bacteriophage tail modules. Tailocins are encoded in the genomes of bacteria by a cluster of genes larger than 40 kbp. The locus includes genes encoding structural proteins, assembly enzymes, chaperones, regulatory genes, and lysis cassettes, which function is to release bacteriocins into the environment [32,34]. Bacteriocins of this type are divided into two groups—R and F. R-type tailocins are evolutionarily related to the tails of phages in the family of *Myoviridae* and form a long shell-encircled tube, at one end of which is a complex basal plate with receptor-binding proteins (RBP) [33]. F-type bacteriocins belonging to the tails of phages in the *Siphoviridae* family do not have a shell. The mechanism of action of tailocins is not fully understood, and presumably involves compression of the shell and penetration of the nucleus through the cell wall, which leads to the formation of a channel or pore that violates the membrane potential of the target cell. The most well-studied phage tail-like bacteriocins are R-and F-pyocins *P. Aeruginosa.* The induction of synthesis of these compounds is associated with the SOS response of the producer cell to DNA damage. The bactericidal spectrum of action of tailocins is usually narrow, and includes subgroups of strains of the producing species [34].

The third type of bacteriocins produced by Gram-negative bacteria is microcins. These are low-molecular weight (<10 kDa), highly stable peptides involved in competitive interactions between members of the *Enterobacteriaceae family.* As a rule, these bacteriocins are resistant to proteases, extreme pH and temperature values [23]. Clusters of genes encoding microcins are located in plasmids, and less often, in genomic DNA. They include a variable number of genes, but demonstrate a conservative organization: open reading frames encoding a microcin precursor, secretion proteins, immune factors, and, in some cases, post-translational modification enzymes [24]. Based on the presence, nature, and localization of modifications, as well as the gene cluster organization and the sequence of leader peptides, microcins are divided into two classes [25].

Class I includes peptides with a molecular weight below 5 kDa (microcins B17, C7-C51, and J25) and complex post-translational modifications. Bacteriocins of this group inhibit vital bacterial enzymes, such as DNA gyrases I and II 68 S and RNA polymerases, and can also have an inhibitory effect on the respiratory chain [26]. Class II includes larger (5–10 kDa) plasmid peptides without post-translational modifications (MccL, MccV, and Mcc24) and linear microcins encoded in the chromosome and carrying a C-terminal siderophore (microcins E492, M, H47, I47, and G47). Their toxic effect is in the formation of pores and destruction of the target cell membrane [35].

Microcins exhibit powerful antibacterial activity, which is based on subtle mechanisms of penetration through the outer and inner membranes of Gram-negative bacteria. To overcome the outer membrane, siderophore-microcins bind receptors involved in iron transport. Cyclic microcin J25, characterized by the presence of an N-terminal macrolactam ring, uses the hydroxomate receptor and the intracellular membrane protein SbmA. Microcin C, produced as heptapeptide adenylate, needs external membrane porines and ABC membrane transporters and turns into a non-hydrolyzable aspartyl-adenylate analog in the cytoplasm [36]. Despite the fact that microcins demonstrate various mechanisms for destroying target cells in the absence of any structural homology, they use a common “Trojan horse” strategy, which makes it possible to use them in the design and development of new effective antibiotics [37].

#### 2.1.2. Bacteriocins from Gram-Positive Bacteria

Most bacteriocins of Gram-positive bacteria refer to two main classes. Class I includes lantibiotics and class II—small post-translationally unmodified bacteriocins. Lantibiotics are peptides that undergo extensive post-translational modifications and contain residues of lanthionine and/or methyllanthionine. In the past decade, it has become clear that this class of ribosomally synthesized and post-translationally modified peptides (RiPPs) is more diverse [17]. The third class of bacteriocins previously included large (more than 10 kDa) antibacterial proteins and bacteriolysins, as well as recently identified tailocins of Gram-positive bacteria [26]. The classification of bacteriocins of Gram-positive bacteria is given in Table 2.

The chemical and spatial structure of bacteriocins and the features of their biosynthesis may differ greatly. 2–21 genetic determinants may be involved in the production of bacteriocins in Gram-positive bacteria. In addition, bacterial strains often produce more than one bacteriocin. Most bacteriocins, with the exception of some classes, are synthesized as a biologically less active precursor with an N-terminal leader peptide attached to the C-terminal peptide of the active center, which is removed by protease to form a mature compound. The leader peptide can also act as a signal. The list of targets and receptors corresponding to bacteriocins is given in Table 3.

Lantibiotics are low-molecular-weight peptides less than 5 kDa in size that contain lanthionine and/or methyllanthionine residues. They give rigidity to the structure of bacteriocin and resistance to the action of proteases. Based on the biosynthesis specifics, lantipeptides are divided into four classes, two of which include compounds with antibacterial activity. Based on the structure features, we can distinguish three types of lantibiotics—AI, AII, and B corresponding to bacteriocins with linear, combined, and globular conformation [63]. AI-type lantibiotics include nisin, epilancin 15X, and microbisporicin. Their antibacterial action is based on inhibition of cell wall synthesis as a result of binding of the bacteriocin N-terminal domain to lipid II, which is a precursor of peptidoglycan. In addition, the C-terminal domain contributes to the formation of pores that lead to violation of the membrane potential. Mersacidin, along with actagardin and cinnamicin is a type-B lantibiotic, with a compact globular tertiary structure. It is assumed that bovicin HJ50 binds to lipid II via an N-terminal fragment, after which the C-terminal domain forms a bond with the membrane, and the disulfide-containing region forms a hairpin-like structure that leads to violation of the bilipid layer integrity [55,64]. A separate group includes two-component lantibiotics that exhibit synergistic antibacterial activity. Lacticin 3147 is the most studied two-component lantibiotic and consists of type B α-peptide (LtnA1) and type A1 β-peptide (LtnA2). The antibacterial effect of lacticin 3147 is also determined by the formation of pores in the target cell membrane [44]. Lantibiotics are of great interest, because of their structural diversity and powerful activity against Gram-positive pathogens. For example, nisin has been used as a natural food preservative for the past 50 years. And several new lantibiotics are currently undergoing clinical trials as antimicrobials [50].

Among the newly discovered groups of lantipeptides also exhibiting antibacterial activity are lipolantins. This class of compounds is characterized by the presence of avionin residue and the N-terminal guanidino fatty acid. The key representative of the group, microvionin is a bacteriocin derived from culture extracts of *Microbacterium arborescens* 5913, active against methicillin-resistant *Staphylococcus aureus* (MRSA) and *Streptococcus pneumonia*. At the moment, the mechanism of action of lipolantins is not fully understood [65].

Class I also includes a group of compounds—thiopeptides that have multiple biological activity (antibacterial, antiviral, antiparasitic, and immunosuppressive). Antibacterial thiopeptides interfere with protein synthesis by binding to the 50S ribosome subunit or elongation factors [42]. Thiopeptides are usually active in the nanomolar range, but their poor water solubility and low bioavailability make it difficult to use them in clinical settings, despite their high activity. Thiopeptide derivative GE2270A is currently the only bacteriocin of this type undergoing clinical trials in the treatment of gastrointestinal infections caused by *Clostridium difficile* [37]. The mannose phosphotransferase system (Man-PTS) is the main mannose permease in bacteria, but it is also a known receptor for class IIa bacteriocins (pediocin-like group), as well as class IId lactococcin A (LcnA) and lactococcin B (LcnB). Class IIa bacteriocins are potent against *Clostridium difficile*, but not against *Lactococcus* spp. In contrast, LcnA-like bacteriocins act only against *Lactococcus lactis* strains. Garvicin Q (GarQ) is a class IId bacteriocin with little similarity to LcnA-like bacteriocins and a relatively broad antimicrobial spectrum, including *Clostridium difficile* and *Lactococcus* spp among others [37].

A group of bacteriocins similar in structure to thiopeptides are modified thiazole/oxazole-microcins-boromycins. Their distinctive features include the presence of macrocyclic amidine, decarboxylated C-terminal thiazole, and several rare β-methylated amino acid residues. The botromycins discovered to date are produced by bacteria of the genus *Streptomyces* spp. and are potent agents against multidrug-resistant microorganisms, such as MRSA and vancomycin-resistant enterococci (VRE). Botromycins also inhibit protein synthesis by interacting with the bacterial 50S ribosomal subunit [45,53].

The family of modified thiazole/oxazole-microcins also includes linear azole-containing peptides (LAPs). At the moment, two compounds of this group—plantazolicin and goadsporin—have been structurally characterized. Plantazolicin is a metabolic byproduct of *B. amyloliquefaciens* FZB42, it shows selective antibacterial activity against closely related strains of the genus *Bacillus.* Goadsporin obtained from *Streptomyces* sp. TP-A0584, also has a narrow spectrum of action, which is limited to members of the genus. The mechanism of action of LAPs has not been studied [38].

Sactibiotics are produced by representatives of the genus *Bacillus* and are unique sulfur-containing peptides that, in addition to antibacterial properties, can also have spermicidal and hemolytic properties. The first compound of this class, subtilosin A, was discovered more than 30 years ago. Sactiotics form rigid hydrocarbon skeleton structures, while the residues of amino acids are located on the surface of the spirals and have a negative charge at a neutral pH, which causes the hydrophobicity of bactericins. The absence of a common cationic charge suggests that sactibiotics are not attracted to the membrane. Combined with a narrow spectrum of action it indicates the interaction of sactibiotics with defined receptors. According to research, subtilosin A uses Trp34 as a membrane anchor and leaves anionic amino acid residues on the surface. Additionally, the peptide can partially embed itself in the membrane and destroy the phosphate head groups and methylene groups of lipids. The destruction of the membrane depends on the concentration of bacteriocin and leads to leakage of vesicular components [49]. Sactipeptides are also referred to as sactibiotics. Sactipeptides are RiPPs bacteriocins containing one or multiple sactionine linkages or sulfur-to-α carbon thiother (sacti) linkages, i.e., crosslinks between the sulfur (S) atom of a Cys and the α-carbon (Cα) of an acceptor amino acid residue [46]. Six sactipeptides are identified so far: subtilosin A, the sporulation killing factor SKF, thurincin H, thuricin CD, the latter being composed of the two distinct sactipeptides Trn-α and Trn-β, huazacin which is also named thuricin Z and finally ruminococcin C. Subtilosin A, Thuricins and SKF are produced by *Bacillus* strains. Very recently, Ruminococcin C was identified as the only sactipeptide identified from a different genus than *Bacillus* so far, indeed, it was isolated from the E1 human fecal strain E1 of *Ruminococcus gnavus* [47]. Importantly, although originally classified as sactipeptides, thermocellin (CteA) and Tte1186a were very recently reclassified as ranthipeptides. 

### 2.2. Structure-Based Classification

#### 2.2.1. Cyclic Structure Classification

The most stable and interesting from the point of view of practical application are bacteriocins with a cyclic structure. This class of compounds also includes lasso peptides, glycocins, and bacteriocins with “head-to-tail” peptide bonds. Lasso peptides got their name due to the tertiary structure specifics, which is determined by the formation of an isopeptide bond between the amine of the N-terminal macrolactam ring and the carboxylic acid residue of glutamic or aspartic acids at positions 7, 8 or 9 of the C-terminal tail peptide sequence. Currently, the structures of three lasso peptide bacteriocins produced by Gram-positive bacteria have been characterized: lariatin A (*Rhodococcus jostii*), streptomonomycin (*Streptomonospora alba)*, and *svicenin* (*Streptomyces sviceus*). Lasso peptides are extremely stable structures that are resistant to the action of enzymes and high temperatures. During linearization, lasso peptides lose their properties and therefore, chemical synthesis of active bacteriocins of this type is not possible. Each lasso peptide is targeted at receptors that are specific to certain genera of bacteria. So, streptomonomycin is a powerful inhibitor of the *Bacillus* species. It is assumed that its bactericidal activity is the result of interaction with the WalR protein involved in cell wall metabolism and division. The highly basic lassomicin affects mycobacteria by inhibiting ATPase. Thus, unlike bacteriocins of other classes that have similar mechanisms of action, due to structural features, the three-dimensional conformation of each lasso peptide imitates a specific signaling molecule [66].

Cyclic bacteriocins, characterized by a bond between the N-and C-termini of the peptide, are called “head-to-tail” and are divided into two subgroups—I with higher (>10) isoelectric point values (pI), and II with a lower pI value. Thermal, pH, and proteolytic stability of ring bacteriocins is explained by the compact three-dimensional structure and cyclization of the termini [39]. Bacteriocins of subgroup I (enterocin AS-48, carnocycline A, and enterocin NKR-5–3B) consist of four to five amphipathic α-helices that are folded into a saposin-like structure. A characteristic feature of the saposin fold is the presence of a V-shaped segment formed by three spirals. The cationic surface charge of bacteriocins of this subgroup provides interaction with target bacterial membranes with subsequent pore formation [48]. Acidocin B belonging to subgroup II is four helices connected by a hydrophobic core. The absence of a saposin fold and the overall neutral charge of this compound suggest a difference in the mechanisms of action of bacteriocins of the two subgroups [67]. 

Gram-positive class II bacteriocins include four groups of compounds—YGNG-motif containing, linear two-peptide, leaderless, and other linear bacteriocins. Bacteriocins of the first group, often referred to in the literature as type IIa or pediocin-like peptides, have several defining structural characteristics, such as a conservative N-terminal YGNG motif, at least one disulfide bridge, an amphipathic α-helix, and a common cationic charge. Peptides show their bactericidal activity by forming pores in the membrane of the target cell. To date, a large number of bacteriocins of this class have been identified, but three-dimensional structures have been determined for only a few of them. The most well-studied compounds are leucocin A (*Leuconostoc gelidum* UAL 187), carnobacteriocin B2 (*Carnobacterium piscicola* LV17), sacacin P (*Lactobacillus sake* Lb706), curvacin A (*Lactobacillus curvatus* LTH1174), and enterocin HF (*Enterococcus faecium M3K31*). They have a distinct S-shaped folded structure at the N-terminus, which is a three-stranded β-sheet forming a hairpin with the help of a disulfide bridge, which is necessary to stabilize the structure. The C-terminus of bacteriocins is involved in the process of interaction with the target cell, which causes the lack of pronounced homology between the compounds and the high specificity of bacteriocins. It is considered that the receptor of this group of peptides is the mannose-phosphotransferase transport complex (Man-PTS). The IIC Man-PTS enzyme contains extracellular loops that interact with bacteriocins, resulting in the penetration of the peptide into the membrane and the formation of pores with subsequent cell death [48,63].

#### 2.2.2. Linear Structure Classification

Linear IIb-class two-peptide bacteriocins are produced as precursors containing double N-terminal glycine-type leader peptides and active center peptides with GXXXG and/or GXXXG-like motifs, where Gly can be replaced by Ala/Ser. The gene cluster responsible for the synthesis of bacteriocin encodes two precursor peptides, immunity protein, ABC transporter, and auxiliary protein. Two-peptide bacteriocins exhibit optimal activity in the presence of equimolar concentrations of both peptides and form pores in the target cell membrane. They are characterized by high conformational lability—in conditions that mimic the membrane, two-peptide bacteriocins become α-helical. Typical antibacterial peptides, such as lactococcin, plantaricin, and carbonobacteriocin, are products of secondary metabolism of lactobacilli and use undecaprenyl-pyrophosphate-phosphotase and amino acid-polyamine-organocationic transporter as target cell receptors [67].

Unlike the previous group, leaderless bacteriocins do not have precursors with an N-terminal leader peptide. As a rule, they contain N-terminal formylmethionine. It has been shown that the presence of a formyl group significantly increases the activity of bacteriocins. Based on differences in three-dimensional structures, this class can be divided into three subgroups—saposin-like, LsbB-like peptides, and laterosporulins. Saposin-like bacteriocins, which include enterocin 7A and 7B, lacticin Q, aureocin A53 and BacSp222, consist of amphipathic α-helices that form a folded structure similar to ring bacteriocins. They are able to enter the cell without receptor’s participation, causing the formation of pores or general destruction of the cell membrane. Typical representatives of LsbB-like peptides are lactococcal small bacteriocin B (LsbB) and enterocin K1. Peptides consist of an N-terminal amphipathic α-helix and an C-terminal random helix. Unlike saposin-like bacteriocins, LsbB and enterocin K1 have narrower action spectra. This group of peptides uses Zn-dependent membrane-bound metallopeptidase as a receptor. Laterosporulins are the only subgroup of leaderless peptides, the members of which undergo posttranslational modifications, such as the formation of a disulfide bond and removal of N-terminal methionine. Currently, this subgroup includes only two peptides—laterosporulin and laterosporulin 10, derived from different *Brevibacillus* sp strains. Structurally, laterosporulins consist of four β-strands in a β-leaf conformation with three disulfide bridges. This structure is close to the mammalian β-defensins and is unusual for bacteriocins. Laterosporulin has been shown to disrupt membrane integrity and reduce ATP levels in target strains, but the details of the mechanism have not been studied [67].

The group of other linear bacteriocins includes single-peptide non-pediocin-like peptides. Lactococcins A and B, enterocin B, carnobacteriocin A, divergycin A, and lactococcin 972 are the most well-studied members of the group. A typical tertiary structure consists of two three-stranded antiparallel β-sheets in a β-sandwich conformation with a predominance of aromatic residues at one terminus. These bacteriocins have a variety of antibacterial spectra, gene cluster organization, primary sequences, secretion mechanisms, and modes of action typical for other groups of this class [68].

The third class of bacteriocins produced by Gram-positive bacteria includes bacteriolysins, non-lytic large bacteriocins, and tailocins. The latter are string-like and functional homologues of phage tail-like bacteriocins of Gram-negative bacteria. These include diphocin, R-type bacteriocin (*Clostridium difficile*), and monocin, F-type bacteriocin (*Listeria monocytogene*).

Bacteriolysins are large polypeptides with a molecular weight of 27 to 35 kDa, which are characterized by sensitivity to higher temperatures and the ability to lyse the cell walls of target bacteria. Bacteriolysins consist of two key domains connected by a linker helix: the N-terminal catalytic domain and the recognition domain at the C-terminus. The catalytic domain is characterized as a hydrolytic protease that targets peptides and peptidoglycan cross-links. In addition to its main function, the substrate recognition domain is also an anchor for the catalytic domain movement along the peptidoglycan chain. The specificity and spectrum of action of bacteriolysins depends on the ability to hydrolyze various peptidoglycan sites. Thus, lysostaphin is metalloprotease, which targets pentaglycine bridge, and the catalytic domain of millericin B is responsible for N-terminal cleavage of both basic peptide glutamine residue and terminal residue of interpeptide cross-linkage. The affinity of bacteriocin to several sites may explain the wider spectrum of action of the compound. The most well-known bacteriolysins produced by Gram-positive bacteria are lysostafin (*Staphylococcus simulans*), zoocin A (*Streptococcus zooepidemicus 4881*), millericin B (*Streptococcus milleri NMSCC 061*), and enterolysin A (*Enterococcus faecalis LMG 2333*) [68].

Non-lytic large bacteriocins are similar in properties to bacteriolysins, but their mechanism of action is not based on cell wall lysis. Examples of such bactericins are helveticin J (*Lactobacillus helveticus 481*), casecin 80 (*Lactobacillus casei*), and dysgalacticin (*Streptococcus dysgalactiae subsp. equisimilis*). Currently, the tertiary structures of these proteins have not been clarified. It is believed that non-lytic bacteriocins block the absorption of glucose and its inclusion in cellular macromolecules. Carbohydrate starvation kills the target cell through a passive non-lytic mechanism [27].

Archaea domain bacteriocins are able to exist in a wide range of habitats, including extreme conditions close to the limit of life. In order to maintain metabolic activity in conditions associated with constant stress, archaea have unique molecular mechanisms for regulating vital activity, a set of in- and out-of-cell enzymes and secondary metabolites. Bacteriocin-like antimicrobial compounds synthesized by archaea are called archaeocins. To date, two main types of archaeocins have been identified—halocins and sulfolobicins produced by *Halobacteriales* (Euryarchaeota) and *Sulfolobales* (Crenarchaeota) archaea [27].

Based on the currently available data, halocins can be divided into two groups of high and low molecular weight. Peptides with a low molecular weight (less than 10 kDa) are usually hydrophobic and more stable than large, sensitive to high temperatures and desalination proteins of the second group. At the moment, only a few halocins obtained from the genera *Haloferax*, *Halobacterium*, and *Natrinema* have been studied, but their characteristics are not complete. The spectrum of action and the degree of activity of halocins depend on the conditions of cultivation and the growth phase of the producer organism. The study and production of antimicrobial compounds is complicated by the difficulties of archaea cultivation and halocin purification, due to the presence of high concentrations of salt in the samples [27].

Producers of the second group of archaeocins are extremely thermophilic acidophiles belonging to the widely studied genus *Sulfolobus.* The most detailed description of this type of antimicrobial compounds was obtained from a study of *S. islandicus* HEN2/2 strain. The activity of the cell fraction in the stationary growth phase is 30 times higher than the supernatant activity. This fact is due to the presence of 50–200 nm spherical vesicles associated with the cytoplasmic membrane, involved in cellular communication and containing quorum-sensitive agents, toxins, pathogenicity factors, DNA and RNA, as well as archaeocins. Three genes that encode sulfasalazine—SulA, SulB and SulC—have been discovered. The genetic organization of loci depends on the strain, but gene clusters are always located in one of two variable regions of the genome, which confirms the similarity with eubacteria bacteriocins, which genes are usually localized near mobile elements or in plasmids [28]. As with most halocins, the culture supernatant containing sulfolobicins is very stable and resistant to extreme environmental conditions. Antimicrobial activity is maintained at a temperature of 78 °C, SDS treatment, long-term storage at a pH in the range of 3 to 10.7. The mode of action of sulfolobicins is still unknown, but it is assumed that they cause growth retardation, rather than lysis of target cells [28].

### 2.3. Sequence-Structure Based Classification

Another type of classification of bacteriocins is the classification based on the sequence-structure relationship [69]. Understanding sequence-structure relationships is important for drug development based on antimicrobial peptides (AMPs). One of the main limitations of AMP databases is the poor use of structural information. The four traditional AMP structures are classified according to secondary structures: α-helical, β-sheet, loop, or extended structures [70,71]. An alternative structural classification using the torsion angles of the peptide backbone also shows many different AMP folds [72]. Some AMP databases, such as APD2, have attempted to link AMP sequences to their secondary structures. Some AMP databases, such as APD2, have attempted to link AMP sequences to their secondary structures. Studying the tertiary structures of AMPs helps to better understand AMPs and accelerate the discovery of potential antimicrobial drugs.

The study [69] presents a database of antimicrobial peptides (ADAM) (available at http://bioinformatics.cs.ntou.edu.tw/ADAM). ADAM comprehensively collects AMP and systematically links AMP sequences and structures. ADAM is integrated from various sources contains the most complete AMP sequences and structures. ADAM not only allows biomedical researchers to search for basic AMP information, but also provides easy access for linking AMP sequences to structures and vice versa.

### 2.4. Classification by Physicochemical Properties of Peptides

Boone et al. [73] developed sets of rules that define physicochemical boundaries that allow direct classification of active sequences and inactive peptides. Existing classification methods are either order insensitive or length dependent, whereas the method presented in [73] generates rulesets that combine order sensitive descriptors with length independent descriptors. This method provides comparable or improved performance to currently available methods. Finding the boundaries of physicochemical properties can lead to new understanding of the similarity of peptides.

## 3. Sources of Bacteriocins

Most of the known bacteriocins most successfully used today are products of secondary metabolism of lactic acid bacteria isolated from milk and containing probiotics of dairy products. LAB are a heterogeneous group of Gram-positive heterotphic bacteria belonging to the phylum *Firmicutes*. This group is particularly interesting because of the long history of safe use of certain lactic acid bacteria and their generally accepted GRAS and QPS safety status. Lactic acid bacteria produce compounds that are part of all three classes of bacteriocins of Gram-positive bacteria. Additionally, much attention is paid to the study of microorganisms that are part of the human and animal microbiota. Most bacteria that inhabit the gut have the ability to produce the bacteriocins necessary to maintain the integrity of the microbial community. As a rule, the spectrum of their action consists of pathogenic enterobacteria and actinomycetes [28].

The most widely studied natural source of bacteriocins is soil. Many bacteriocins extracted from rhizosphere and soil bacteria are used for plant protection. Thus, *Pseudomonas putida* BW11M1, isolated from basal microbial communities of banana, produces putidacin, which destroys the plant pathogen *P. putida* GR12-2R3. Other examples include bacteriocin Bac 14B (*B. subtilis* 14B), which is effective against the causative agent of a disease caused by *Agrobacter tumefaciens*, and Bac GM17 (*B. clausii* GM17), which has broad-spectrum antibacterial and antifungal activity. In addition, some plant pathogens produce antibacterial substances. The phytopathogenic strain *Erwinia carotovora* NA4 isolated from affected vegetables and fruits produces euriniocin NA4, a pathogen of tomato *Clavibacter michiganensis* ssp. *Michiganensis*—bacteriocin michiganin A, which inhibits the growth of *C. michiganensis* subsp. *Sepedonicus*, which causes ring rot of potatoes. Most of the soil bacteriocins are synthesized by representatives of the genus *Bacillus* and are actively used as bioinsecticides and biopesticides, as well as growth stimulants [28].

One of the most interesting producers of bacteriocins from the point of view of fundamental and applied science are marine bacteria and strains that make up microbial communities of sediments. Less attention is paid to these bacteriocins, but they are highly diverse and have significant potential for use in biotechnology, primarily as probiotics and antibiotics in the fishing industry and marine aquaculture [41]. The first marine bacteriocin was isolated from *Vibrio harveyi* after screening 795 strains of *Vibrio* species isolated near Galveston island (Texas, USA). The identification of harveycin has become the starting point for numerous studies focused on the biochemical characterization of new bacteriocins and bacteriocin-like compounds. To date, it is known that approximately 10% of marine bacteria that form biofilms have antibacterial activity [17]. Produced bacteriocins can be small peptides (5–10 kDa), such as microcins of Gram-negative bacteria, microgalocins of halobacteria, and class I and II bacteriocins of Gram-positive bacteria, as well as larger compounds (10–90 kDa), such as colicins and colicin-like bacteriocins of Gram-negative bacteria. The main genera of marine bacteria that produce bacteriocins are *Aeromonas*, *Burkholderia*, *Photobacterium*, *Bacillus*, *Pseudomonas*, *Serratia*, *Stenotrophomonas*, *Carnobacterium*, *Lactococcus*, *Streptomyces*, *Pseudoalteromonas*, *Enterococcus*, *Pediococcus*, as well as some archaea. The main difference of marine bacteriocins is their high resistance to high and low temperatures, osmotic stress, and various proteolytic enzymes and organic solvents [28]. 

Regardless of the source, obtaining pure bacteriocin is a fairly complex process of screening and cleaning the compound. The most common traditional methods of testing antibacterial activity are spot analysis on a lawn, disc diffusion and diffusion analyses [41]. In this case, indicator strains that are in the exponential growth phase are inoculated into appropriate agar culture media or distributed in a cup with a dense culture medium. The most commonly used indicator strains are *E. coli* and *S. aureus*, but *Listeria monocytogenes* and *Micrococcus luteus* are also used. Since spot analysis on a lawn requires a test sample of at least 10 µL, this method is most suitable for substances with strong antibacterial activity, such as purified extracts. For agar diffusion analysis and the disc diffusion method, approximately 100 µL of sample is required to fill the well or soak the disc. Thus, these methods are suitable for low-activity supernatant samples that can be easily obtained by centrifugation. For all three methods, the antimicrobial activity of bacteriocin is evaluated by measuring the lysis zone around the agent application point. This makes it easier to visualize the results, but it can take a long time [41]. 

Since thousands of strains are screened when searching for new compounds, the efficiency and accuracy of the analysis are the most important factors. One of the methods that meets these criteria is PCR analysis, making it possible to quickly and easily identify the presence of bacteriocin-encoding genes. The bacteriocin PCR matrix is based on known bacteriocin-related enterococcal genes from the NCBI GenBank database. To date, this method is actively used for screening of bacteria that produce lantibiotics. However, it is worth noting that different producing strains usually have different gene sequences, and PCR analysis of the selection of primer pairs for each of them. There is also an in vivo screening method that involves creating a platform using 96-well tablets and fluorescent markers to identify bacteriocins from natural sources. Currently, this method is used to search for compounds that are active against *S. aureus.* In vivo screening is considered to be the fastest, most reliable and cost-effective method compared to traditional methods [41].

The identification of bacteriocin-producing strains uses physiological, biochemical criteria, and analysis of 16S rRNA genes. It is also important to study the supernatant at different pH values, temperatures, and enzyme resistance. Currently, there are several databases of antimicrobial compounds. The APD antimicrobial peptide database contains information on 2619 antimicrobial compounds and 261 bacteriocins and includes producer organisms, peptide sequences, chemical modifications, structure and additional functions of peptides, target organisms, mechanism of action, year of discovery, and author. In addition, the APD peptide properties calculator allows researchers to calculate the molecular weight, molecular formula, and molar extinction coefficients of bacteriocins. The collection of antimicrobial peptides (CAMP) [43] is a database of antimicrobial peptides that contains their sequences and structures and is linked to other databases, such as AMPer, focused on natural bacteriocins and CyBase, which includes cyclic polypeptides and proteins. BACTIBASE is the most frequently used database [43]. It includes various tools for bacteriocin analysis, including homology search, multiple sequence alignments, HMM analysis, molecular modeling, and taxonomy search [43]. The development of new bioinformatic tools will simplify the identification and annotation of new bacteriocins or bacteriocin-like proteins.

After screening and identification of new bacteriocins, the most important and complex stage is purification. Currently, the choice of purification method is determined by the molecular weight, charge and properties of bacteriocin. The simplest method used to produce enterocin L11 includes ethanol extraction and cross-linked glucosane chromatography. Complex purification processes involve ammonium sulfate precipitation, cation exchange, gel filtration chromatography (GFC), and high-performance liquid phase chromatography (RP-HPLC). Most of the bacteriocins detected over the past 5 years were obtained by ammonium sulfate precipitation, ion exchange chromatography (IEC), or RP-HPLC. However, since there is no universal method, the process completely depends on the characteristics of the bacteriocin of interest [39].

Usually, bacteriocins are obtained by culturing the producing strain, but there are also examples of attempts to produce recombinant compounds. Their synthesis is carried out by expression in heterologous systems, but the activity of such bacteriocins is very low or absent. In addition, due to the low efficiency of microbial fermentation, an alternative approach was developed using solid-phase peptide chemical synthesis (SPPS). Lower prices for reagents and “building blocks” used for peptide synthesis, and advances in post-translational modification catalysis to produce native proteins have made chemical synthesis an attractive and competitive alternative approach for biomedical applications. At the moment, bacteriocin AS-48, uberolisin, garvicin ML and pediocin PA-1 have been successfully synthesized. The developed bioinformatic program Word2vec makes it possible to analyze a section of a peptide with a length of 20 amino acids based on three properties: charge, corformation and hydrophobicity. As a result of scanning 676 different putative sequences of bacteriocins, 16 artificial peptides were synthesized that showed high antimicrobial activity against *E. coli* and *P. aeruginosa*. The disadvantage of this method is low productivity which requires further optimization of the process to increase production profitability [39].

Currently, bacteriocins are widely used in the food and pharmaceutical industry, medicine, seafood industry and agricultural processes.

Bacteriocins synthesized by lactic acid bacteria are currently the only ones used for food preservation and have many advantages over antibiotics or food preservatives obtained by chemical synthesis. Pure and mixed cultures of lactic acid producing bacteria, as well as bacteriocin prepatars, such as nisin, acidocin and propionicin, are actively used as antibacterial agents against bacteria and pathogens that cause food spoilage and are applied to preserve and stabilize various types of food, including fermented milk products, mayonnaise spreads, cream, cheese products, meat, or vegetable compositions. In addition, the use of such cultures can improve the quality of food products and their organoleptic properties [61].

The use of starter cultures that produce bacteriocins in the dairy industry is beneficial for protecting fermented foods from the transmission of food pathogens. Secondary cultures with bactericidal potential can also help accelerate the maturation of dairy products. For example, the addition of lactococcin ABM promotes early lysis and release of intracellular enzymes from the starter culture and promotes early maturation of cheese. The use of purified and concentrated bacteriocins as food supplements is more preferable than the use of cultures with the potential to produce bacteriocins, due to the slow growth of bacteria or the production of bacteriocins at a late stage of the life cycle [58].

Wheat dough used for the preparation of various bakery products is also susceptible to contamination by bacteria, which leads to defects in bread, including an increased content of enterotoxins. In low concentrations, bacteriocins are effective against vegetative cells of the strains *B. subtilis*, *B. licheniformis, B. cereus*, and *B. pumilus*, while higher concentrations (23 u/g) are necessary for the destruction of endospores. Bacteriocin AS48 is effective against *S. aureus*, *B. cereus*, and *L. monocytogenes* when added to baking cream, soy desserts, and gelatin puddings. The gelatinous form of pediocin, a IIa class bacteriocin, is used to protect hot dogs from *Listeria* infection. Strains of *L. monocytogenes* are widely distributed in nature and are also found in ready-to-eat meat. It is known that bacteriocins of lactic acid bacteria reduce or inhibit their growth during meat product processing [56,58].

Most fresh vegetables and fruits are consumed raw, which carries risks to human health if they are contaminated with pathogenic bacteria. 

## 4. Antimicrobial Activity of Bacteriocins

The use of bacteriocins can prevent the spread of pathogenic microorganisms. Thus, treatment with enterocin AS-48 inactivates *L. monocytogenes* cells inoculated on the fruit surface and slices, and partially or completely inactivates *S. Aureus* in various vegetable and fruit sauces [12]. Bovicin HC5, kills spores of the heat-stable *Alicyclobacillus acidoterrestris*, the main bacterium that causes spoilage of pasteurized acidic juices, and spores of *Geobacillus stearothermophilus* in coconut milk. The use of bacteriocins, including the addition of such antibacterial substances as sodium perchlorate, can significantly increase the shelf life of fresh vegetables and fruits and foods produced from them [44]. The growth of undesirable microorganisms can also worsen characteristics of alcoholic beverages. Bacteriocins of lactic acid bacteria, such as nisin, are used to reduce the level of sulfur dioxide in wine. Compositions prepared by fortifying the wort with a food additive containing a culture that produces nisin provide high stability during storage of beer without changing its taste and aroma [67].

Spray-dried lacticin 3147 powder has effective antimicrobial activity, and can be used in a number of food products, such as baby milk, powdered soup, yogurt, and cottage cheese [32]. It is shown that lacticin is non-toxic and non-immunogenic, and also suppresses the development of infections. It is important to use lactic acid bacteria (*Lactococcus lactis* 3147) that produce lacticin, as they inhibit the growth of many types of Gram-positive microorganisms, including *Listeria monocytogenes*, *Clostridium botulinum*, *Clostridium sporogenes*, *Staphylococcus aureus*, *Pediococcus acidilactici*, *Bacillus* spp., *Enterococcus* faecalis [16]. The significance of using biologically active powder is that it can be easily used as an ingredient in various food products [67].

In animal farming, bacteriocins also play an important role in controlling the overgrowth of potentially pathogenic bacteria. The lack of maternal bacterial flora or the induction of a proper immune system in newly hatched broiler chickens makes them susceptible to infection [15]. The use of *E. faecium* bacteriocins after hatching increases the survival rate of chickens infected with the poultry pathogen *S. Pullorum* and *E. coli* microcins contribute to the destruction of *S. typhimurium* in adult chickens. There are reports that the introduction of colicin-producing bacteria into the rumen of cows reduces the number of intestinal pathogens in animals [44]. As a rule, probiotic mixtures based on sorbic acid and bacteriocins or bacteriocin-producing crops are used in animal farming. These mixtures are used as additives in feed and drinking water [73].

The most important applications of bacteriocins are related to human health. One of these areas is the use of bacteriocin producers as probiotics. Probiotics are non-pathogenic and non-toxic strains, beneficial to the host animal, that are able to survive and maintain metabolic activity in the intestinal environment and remain stable and viable for long storage periods [26]. Probiotics demonstrate the potential for antimicrobial production, competitive pathogen destruction, competition for nutrients, and immune system modulation. Many antibacterial substances, such as bacteriocins, short-chain fatty acids, and hydrogen peroxide are produced by probiotics to inhibit gastrointestinal pathogens. Currently, many probiotics are used in everyday life, including lactic acid bacteria, non-pathogenic strains of *E. coli*, *Bacilli*, and yeast [68].

It has been shown that purified bacteriocins or bacteriocin-producing probiotics can reduce the number of pathogens or change the composition of the intestinal microbiota in mice, chickens and pigs [17]. A strain of *Lactococcus lactis* that produces nisin to stimulate the growth of Bifidobacterium in the intestines of rats and suppress the reproduction of enterococci and streptococci in the duodenum, ileum, caecum and colon [45]. Colicin Ib, E1 and microcin C7, derived from the *E. coli* H22 strain have the ability to inhibit the growth of pathogenic and non-pathogenic bacteria, such as *Enterobacter*, *Escherichia, Klebsiella*, *Morganella*, *Salmonella*, *Shigella*, and *Yersinia.* It has also been demonstrated that probiotic strains of human origin, such as *Lactobacillus salivarius* UCC118, produce bacteriocin Abp118 that can destroy *Listeria monocytogenes* cells [33]. *Pediococcus* spp. lactic acid bacteria are also widely described as probiotics. There are many strains of *Pediococcus* that produce pediocin, an effective agent for killing *Listeria*. Recent research in this area is aimed at a full-scale study of probiotics, in order to use them in the pharmaceutical and food industries [27,68].

Currently, it is proposed to use bacteriocins as an alternative to antibiotics [28]. The narrow spectrum of action allows using bacteriocins as highly specific antibacterial compounds that target specific bacterial pathogens. Given the variety of natural bacteriocins, it is easy enough to find drugs among them that are active against specific human pathogens. The development and use of such narrow-spectrum antimicrobials will not only increase the number of available drugs, but also extend the life of classic antibiotics [41].

The main research is focused on the possibility of using bacteriocins of Gram-negative bacteria, primarily colicins and pyocins. While the data on their use as antibiotics obtained in the past few years from in vivo experiments is encouraging, there are key questions regarding their suitability as therapeutics that have yet to be resolved. First, only preliminary data are available on the effect of bacteriocins on the patient in terms of toxicity or immune response [40]. However, the available information does not report negative or toxic effects against the body. The exception is the mortality of chickens treated with pyocins, but it is not known whether the preparation was cleared of endotoxins. In addition, additional studies of dosage regimens and the time of bacteriocin administration in particular are needed. To solve this problem, it is necessary to conduct more detailed pharmacokinetic studies [54].

Comparing the effectiveness of bacteriocins to the effectiveness of conventional antibiotics has led to encouraging results [15]. Piocin S5 is at least 100 times more effective than tobramycin in treating lung infection in mice, making it a promising replacement for traditional antibiotics used against *P. aeruginosa.* However, most bacteriocins are characterized by low stability and must be used in higher doses with short intervals between repeated doses [43]. However, low stability can also reduce the impact of the antibiotic on the body and the environment, which minimizes resistance development. As a result of research, it was found that resistance to bacteriocins in the environment can occur through the modification of cell surface receptors [39]. It is worth noting that, despite the possible appearance of resistance, many bacteria remain sensitive to certain concentrations of bacteriocins [61].

The disadvantage of using bacteriocins may be the natural immunity of producing strains. The presence of common target cell destruction mechanisms and identical cytotoxic domains can lead to cross-immunity between bacterial strains [64]. However, most bacterial strains do not express immune proteins, or produce one [45]. Thus, bacteria can only be immune to a limited number of bacteriocins. It is assumed that the use of a cocktail of bacteriocins will bypass cellular immunity [58].

## 5. Bacteriocins in Cancer Treatment

The cytotoxicity of bacteriocins and their ability to affect cancer cells may depend on structural properties such as the number of positively charged amino acids, hydrophobicity, and the ability to form amphipathic structures and oligomers. It is assumed that the main target for bacteriocins is the cytoplasmic membrane of eukaryotic cells. It is established that bacteriocins cause the death of organisms close to the organism producing these bacteriocins [16]. Bacteriocins have a relatively narrow spectrum of action, because they are active against bacteria of the same or phylogenetically related species, which are cancer cells. The action of bacteriocins is selective, and they are able to specifically bind only to cancer cells [16]. Increasing the expression of negatively charged molecules on the surface of cancer cells makes them vulnerable to the cytotoxic activity of bacteriocins [56]. Cytotoxicity mechanisms include induction of apoptosis and/or depolarization of the cell membrane, which leads to changes in permeability. Some of them can cause both necrosis and apoptosis. Studies measuring the membrane potential demonstrate that, within a few seconds after the interaction of cytotoxic bacteriocin with a sensitive eukaryotic cell, its surface is depolarized and its permeability increases, leading to cell death. Rapid destruction caused by cytotoxic bacteriocins may indicate a non-receptor mechanism of action [55].

Despite the fact that the cytotoxic potential of bacteriocins has been studied in the laboratory, there is no data on the effectiveness for cancer treatment. The advantages of bacteriocins as therapeutic agents are that they are small peptides and thus, for the most part, are not immunogenic in nature. Second, they are easily hydrolyzed to simple amino acids [59]. One of the main problems of using bacteriocins as medicines is the reduction of stability in the intestines or human tissues. Attempts have been made to chemically synthesize peptides that include D-amino acids, which are less susceptible to proteolytic cleavage in the gut. Analogs of lactococcin G were synthesized with N-and C-terminal residues replaced with D-amino acids that have a reduced sensitivity to the action of exopeptidases without significant effect on activity. Directed changes in trypsin recognition sites in salivaricin P resulted in a stable compound with a slight change in its activity. Such studies aimed at improving the stability and effectiveness of antitumor bacteriocins are justified. In addition, functional carriers for directed and controlled delivery of bacteriocins can also improve their stability in vivo [57].

The integration of nanotechnologies and biotechnologies opens the way to unlimited opportunities and future prospects for solving problems related to a range of biological products. Through this integration, effective delivery, targeting, protection from degradation, as well as increasing the effectiveness of drugs can be achieved [60]. Nanoincapsulation of bacteriocins intended for use as bioconservants can protect them from degradation by proteolytic enzymes and prevent undesirable interactions with other food components. In addition, some recent studies show that encapsulation of bacteriocins in nanoparticles increases their activity against microorganisms that cause spoilage of products and multidrug-resistant bacteria. In addition, the use of materials and/or methods based on nanotechnology, in most cases, has a positive effect on the production of bacteriocins [35,74].

It is worth noting that due to their antimicrobial and antifungal properties, nanoparticles have the ability to inhibit the growth of numerous microorganisms. Nanoparticles come into contact with the surface of a pathogenic bacteria cell. This interaction leads to structural changes and inhibition of vital functions of the cell, leading to its death. The use of bacteriocin nanoconjugates has a high potential in the food industry, contributes to an increase in the antimicrobial spectrum of compounds and can reduce the need for a high bacteriocin dosage and prolong the shelf life of food. Various approaches are used for the formation of conjugates, including the encapsulation of bacteriocins in nanoliposomes, the conjugation of bacteriocins with chitosan nanoparticles, and the interaction of bacteriocins with metal nanoparticles [74].

However, it is necessary to find out the nature of interactions between antibacterial peptides and nanomaterials, between bacteriocin nanoconjugates and target microorganisms, and to evaluate the effectiveness in vivo and the safety of the compounds. Understanding these features opens up prospects for clinical use of bacteriocins in the near future [62].

## 6. Nanotechnologies in the Use of Bacteriocins

According to the Science and Technology Committee of the House of Lords of the United Kingdom, nanotechnology is the transformation of functional materials and structures into nanoscale sizes (with diameters from 1 to <1000 nm) [75]. This is a completely new technology that has several applications in various fields of science, due to the unique properties of synthesized nanoparticles [76]. The integration of nanotechnology and biotechnology opens the door to unlimited possibilities and future prospects for solving the problems associated with a number of biological products. Through this integration, efficient delivery, targeting, protection from degradation, as well as improved drug activity and physicochemical properties, can be achieved [77]. Bacteriocins are one of the many examples that can benefit from this combination. For example, nanoencapsulation of bacteriocins intended for use as bio-preservatives can protect them from degradation by proteolytic enzymes, as well as save them from unwanted interactions with other food components and, therefore, increase their stability for longer periods [78]. In addition, some recent studies have shown that the encapsulation of bacteriocins in nanoparticles enhanced the activity of these peptides against food spoiling microorganisms and multidrug resistant bacteria [79,80]. Moreover, the use of materials and/or methods based on nanotechnology in most cases showed a positive effect on the yield of bacteriocins, which facilitated their commercial production [81].

Recent findings indicated that bacteriocins might be used to prevent bacterial infections in vivo and may also function as immune modulatory agents. Although limited, some reports suggested that bacteriocins may be used in the treatment of high blood pressure and even control the growth of tumors. However, most bacteriocins are unstable in vivo, and need to be protected from proteolytic enzymes and the body’s natural defense mechanisms. This pushed research on bacteriocins into a new direction, nanotechnology [82]. The incorporation of bacteriocins into nanoparticles and site-directed delivery to areas of infection may soon become an effective method of treatment. Delivery of bacteriocins in a concentrated form, such as encapsulated into nanoparticles, would increase their effectivity and minimize possible toxic side effects. This work reviews the latest development in peptide nanotechnology, and addresses some of the problems encountered in the design of target-specific bacteriocin delivery systems [82].

## 7. Conclusions

The paper summarizes information about the types and classes of bacteriocins, describes methods for their preparation and purification through PCR reactions, and shows the probiotic and antibacterial effect of bacteriocins on human and animal bodies (a method for studying gastrointestinal microbiota). The main conclusion from the data obtained is to establish the cytotoxicity of bactcriocins, i.e., the ability to toxically affect cancer cells and destroy them. These findings require additional research, including clinical studies on cancer patients, but the prospects and relevance of this area of science are not in doubt. It is established that incorporation of bacteriocins into nanoparticles and site-directed delivery to areas of infection may soon become an effective method of treatment. The delivery of bacteriocins in a concentrated form, such as encapsulated into nanoparticles, would increase their effectivity and minimize possible toxic side effects.

The search for new bacteriocin-producing strains with unique properties and characteristics can expand the area of their industrial application and open up new prospects for use.

Due to the wide variety of bacteriocins, high cytological flexibility, direction of action, their real potential has not yet been fully evaluated. Researchers working with bacteriocins have the following main tasks:

- Search for new bacteriocin-producing strains;
- Improve the productivity of strains (by selection and genetic engineering);
- Search for strains with new properties;
- Study the effect of production conditions on the content of bacteriocins;
- Optimization of production processes (cost reduction and increased product yield);
- Environmentalization of production (introduction of closed production cycles, reduction of waste, the most comprehensive use of components);
- Assessment of environmental, economic, medical risks from scaling up production.

Multifaceted and joint work on the solution of these problems will make it possible to replace the currently existing unstable production processes with alternative, less destructive ones. Innovations optimizing the production of bacteriocins will make their use economically feasible and marketable in the future.

## Figures and Tables

**Table 1:** Classification of bacteriocins.

Bacteria Name	Class	Size (kDa)	Examples	Source
Gram-negative bacteria	Colicins	30–80	Colicins A, B, E2, E3	[17,18,19]
	Colicin-like bacteriocins	30–80	S-piocins, klebicins	[17,19,20]
	Bacteriocins, phage-tail like	20–100	R- and F-piocins	[17,19,21]
	Microcins	<10	Microcin C7, microcin B17, colicin V	[17,19,22]
Gram-positive bacteria	Class I	<5	Nisin, mersadicin, lacticin 3147	[19,23]
	Class II	<10	Pediocin RA1, carnobacteriocin B2	[19,24]
	Class II	>10	Helvecin, enterocin AS-48	[19,25,26]
Archaea	Halocins	>5	Halocin A4, C8, H1, H4	[19,27]
	Sulfolobaceae	~20	Sulfolobaceae	[19,28]

**Table 2:** Classification of bacteriocins of Gram-positive bacteria.

Class	Group	Distinctive Characteristic	Source of Information
Class I	Lantibiotics	Residues (methyl)lanthionine	[17,38,39]
	Lipolantins	N-terminal fatty acid and avionin fragment	[26,27,29]
	Thiopeptides	6-membered nitrogen heterocycle, azole rings	[26,28,40]
	Botromycins	Macrocyclic amidine, decarboxylated C-terminal thiazole, β-methylated residues	[26,28,39,41]
	Linear azole-containing peptides	thiazole and (methyl)oxazole rings, linear back bone	[19,42,43]
	Sactibiotics (sactipeptides)	Disulfide α-carbon bridges	[19,44,45,46,47]
	Lasso peptides	Cyclization of an N-terminal amine into a γ-acid	[17,27,40]
	Cyclic bacteriocins with a “head-to-tail” connection	Cycling from N-terminus to C-terminus	[26,27,48]
	Glycocins	Glycosylated residues	[17,37]
Class II	YGNG-motif containing bacteriocins	Consensus YGNG-motif, at least one disulfide bridge	[19,48,49]
	Linear two-peptide bacteriocins	Synergy of two peptides	[19,28,49]
	Leaderless bacteriocins	Lack of a leader peptide	[26,43,50]
	Other linear bacteriocins	Non-YGNG-like linear peptides	[19,42,51]
Class III	Bacteriolysins	Large lytic polypeptides,	[27,52,53]
	Non-lytic bacteriocins	Large non-lytic polypeptides	[41,49,52]
	Tailocins	Multiprotein complex, a structure similar to a phage tail	[21,53,54]

**Table 3:** Targets and receptors of bacteriocins of Gram-positive bacteria.

Target	Bacteriocin	Group	Source of Information
Lipid II	Nisin, microbisporicin, bovicin HJ50, mersacidin, lacticin 3147, haloduracin	Lantibiotics	[52,55]
	Lactococcin 972	Other linear bacteriocins	[52,56]
Phosphatidylethanolamine	Cinnamycin	Lantibiotics	[52,57]
50S ribosomal subunit	Thiostrepton, nosiheptide, micopoccine	Thiopeptides	[52,58]
	Botromycin A2	Botromycins	[52,56]
Extension factor TU	Thiopeptide GE2270A	Thiopeptides	[52,56,58]
Regulator of cellular response WalR	Streptomonomicin	Lasso peptides	[57,59]
ClpC1 ATPase	Lassomycin	Lasso peptides	[35,60]
Glucose-phosphotransferase system	Sublancin 168, glycocin F	Glycocins	[35,60]
MscL mechanosensitive channel	Sublancin 168	Glycocins	[55,61]
Maltose ABC-transporter	Garvicin ML	Cyclic bacteriocins with a “head-to-tail” connection	[52,60,61]
Mannose-phosphotransferase system	Pediocin PA-1, leucocin A, carnobacteriocin B2, sacacin P, curvacin A, enterocin HF	YGNG-motif containing bacteriocins	[56,60]
	Lactococcin A, garviacin Q	Other linear bacteriocins	[58,60,61]
Undecaprenyl-pyrophosphate-phosphatase	Lactococcin G, enterocin 1071	Two-peptide bacteriocins	[39,58]
Amino acid-polyamine-organocation transporter	Plantaricin JK	Two-peptide bacteriocins	[35,39,55]
Zn-dependent membrane-bound metallopeptidase	LsbB, enterocin K1	Leaderless bacteriocins	[58,62]
Peptidoglycan	Lysostaphin, zoocin A, millericin B, enterolisin A	Bacteriolysins	[52,56,58]
Lipopolysaccharides	Diffocin, monocin	Tailocins	[35,39,52,56]
